# Impact and Outcomes of an Iyengar Yoga Program in a Cancer Centre

**DOI:** 10.3747/co.v15i0.284

**Published:** 2008-08

**Authors:** M.D. Duncan, A. Leis, J.W. Taylor–Brown

**Affiliations:** Department of Community Health and Epidemiology, University of Saskatchewan, Saskatoon, Saskatchewan, and Department of Patient and Family Support Services, CancerCare Manitoba, Winnipeg, Manitoba, Canada

**Keywords:** Iyengar yoga, cancer, complementary and alternative medicine, integrative oncology, mixed methodology

## Abstract

**Background:**

Individuals have increasingly sought complementary therapies to enhance health and well-being during cancer, although little evidence of their effect is available.

**Objectives:**

We investigated

- how an Iyengar yoga program affects the self-identified worst symptom in a group of participants.
- whether quality of life, spiritual well-being, and mood disturbance change over the Iyengar yoga program and at 6 weeks after the program.
- how, from a participant’s perspective, the Iyengar yoga program complements conventional cancer treatment.

**Patients and Methods:**

This pre–post instrumental collective case study used a mixed methods design and was conducted at a private Iyengar yoga studio. The sample consisted of 24 volunteers (23 women, 1 man; 88% Caucasian; mean age: 49 years) who were currently on treatment or who had been treated for cancer within the previous 6 months, and who participated in ten 90-minute weekly Iyengar yoga classes.

The main outcome measures were most-bothersome symptom (Measure Your Medical Outcome Profile 2 instrument), quality of life and spiritual well-being (Functional Assessment of Chronic Illness Therapy–General subscale and Spiritual subscale), and mood disturbance (Profile of Mood States–Short Form). Participant perspectives were obtained in qualitative interviews.

**Results:**

Statistically significant improvements were reported in most-bothersome symptom (*t*_(23)_ = 5.242; *p* < 0.001), quality of life (*F*_(2,46)_ = 14.5; *p* < 0.001), spiritual well-being (*F*_(2,46)_ = 14.4; *p* < 0.001), and mood disturbance (*F*_(2,46)_ = 10.8; *p* < 0.001) during the program. At follow-up, quality of life (*t*_(21)_ = −3.7; *p* = 0.001) and mood disturbance (*t*_(21)_ = 2.4; *p* = 0.025) significantly improved over time. Categorical aggregation of the interview data showed that participants felt the program provided them with various benefits not included on the outcomes questionnaires.

**Conclusions:**

Over the course of the Iyengar Yoga for Cancer program, participants reported an improvement in overall well-being. The program was also found to present participants with a holistic approach to care and to provide tools to effectively manage the demands of living with cancer and its treatment.

